# HELP THAT HURTS: HOW BENEVOLENT AGEISM AND ABLEISM INTERSECT

**DOI:** 10.1093/geroni/igae098.4204

**Published:** 2024-12-31

**Authors:** Devin Thompson, Carla Strickland-Hughes

**Affiliations:** University of the Pacific, Modesto, California, United States; University of the Pacific, Stockton, California, United States

## Abstract

Older adults and people with disabilities are common victims of overaccommodative and unwanted help, partially due to their shared stereotypes of incompetence. This research replicated past work on perceptions of benevolent ageism (Chasteen et al., 2021), extending with examination of ableism and ratings of need for help. We aimed to test how younger and older persons’ impressions of an older woman (e.g., warmth) were affected by her reaction to benevolent ageism, depending on her physical disability. Younger (n=205; 18-35 years old) and older (n=216; 55+ years old) participants were recruited via MTurk, CloudResearch, and participant pools. Participants were randomly assigned to read a benevolent ageism scenario where an older woman had a cane or not and reacted by politely confronting or reluctantly accepting unnecessary help. Participants rated the older woman before and after her reaction. Consistent with preregistered hypotheses and past research, ratings of warmth, competence, and overall impression increased whereas ratings of perceived need for help decreased when the older women moderately confronted the offer of help. These effects were more pronounced for older participants than younger participants. However, the presence of the cane influenced impressions by younger, but not older, participants, who rated the older women as more warm after confronting ageism only when she had a cane. This age difference in perception based on the cane might be explained by experience with mobility aids. Implications of results for understanding how ableism and ageism interact for perceivers of different ages and abilities will be discussed.

